# 2,4-Bis(4-fluoro­phen­yl)-1,5-dimethyl-3-aza­bicyclo­[3.3.1]nonan-9-one

**DOI:** 10.1107/S1600536813024689

**Published:** 2013-09-12

**Authors:** S. Rizwana Begum, R. Hema, R. Venkateswaramoorthi, K. Krishnasamy, A. G. Anitha

**Affiliations:** a Department of Physics, Seethalakshmi Ramaswami College (Autonomous), Tiruchirappalli 620 002, India; b Department of Physics, K. Ramakrishnan College of Engineering, Samayapuram, Tiruchirappalli 621 112, India; c Department of Chemistry, Annamalai University, Annamalai Nagar 608 002, India

## Abstract

The asymmetric unit of the title compound, C_22_H_23_F_2_NO, contains two independent mol­ecules, *A* and *B*. The bicyclic system adopts a twin-chair conformation in both mol­ecules. The dihedral angles between the fluoro­phenyl rings are 55.27 (8) and 56.37 (7)° in mol­ecules *A* and *B*, respectively. The NH groups are not involved in hydrogen bonding due to the steric hindrance of fluoro­phenyl groups. The crystal structure features weak C—H⋯O inter­actions.

## Related literature

For related structures, see: Venkateswaramoorthi *et al.* (2013 ▶); Pham *et al.* (1998 ▶). For the synthesis of 1,5-dimethyl-2,4-diphen­yl-3-aza­bicyclo­[3.3.1]nonan-9-one derivatives, see: Venkateswaramoorthi *et al.* (2012 ▶). For ring puckering parameters, see: Cremer & Pople (1975 ▶).
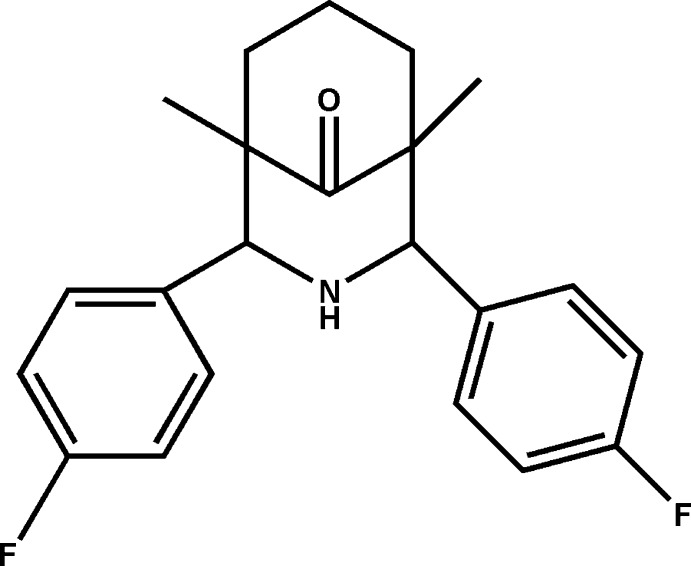


## Experimental

### 

#### Crystal data

C_22_H_23_F_2_NO
*M* *_r_* = 355.41
Monoclinic, 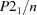

*a* = 8.8470 (3) Å
*b* = 20.5656 (8) Å
*c* = 20.6403 (9) Å
β = 98.633 (2)°
*V* = 3712.8 (3) Å^3^
*Z* = 8
Mo *K*α radiation
μ = 0.09 mm^−1^
*T* = 293 K
0.35 × 0.35 × 0.30 mm

#### Data collection

Bruker Kappa APEXII CCD diffractometer
Absorption correction: multi-scan (*SADABS*; Bruker, 2004 ▶) *T*_min_ = 0.969, *T*_max_ = 0.973
32409 measured reflections
6363 independent reflections
3974 reflections with *I* > 2σ(*I*)
*R*_int_ = 0.038

#### Refinement

*R*[*F* ^2^ > 2σ(*F* ^2^)] = 0.043
*wR*(*F* ^2^) = 0.125
*S* = 1.01
6363 reflections
473 parameters
3 restraints
H-atom parameters constrained
Δρ_max_ = 0.12 e Å^−3^
Δρ_min_ = −0.28 e Å^−3^

### 

Data collection: *APEX2* (Bruker, 2004 ▶); cell refinement: *APEX2* and *SAINT* (Bruker, 2004 ▶); data reduction: *SAINT* and *XPREP* (Bruker, 2004 ▶); program(s) used to solve structure: *SIR92* (Altomare *et al.*, 1993 ▶); program(s) used to refine structure: *SHELXL97* (Sheldrick, 2008 ▶); molecular graphics: *ORTEP-3 for Windows* (Farrugia, 2012 ▶); software used to prepare material for publication: *SHELXL97*.

## Supplementary Material

Crystal structure: contains datablock(s) I, global. DOI: 10.1107/S1600536813024689/bh2483sup1.cif

Structure factors: contains datablock(s) I. DOI: 10.1107/S1600536813024689/bh2483Isup2.hkl

Supplementary material file. DOI: 10.1107/S1600536813024689/bh2483Isup3.cml

Additional supplementary materials:  crystallographic information; 3D view; checkCIF report

## Figures and Tables

**Table 1:** Hydrogen-bond geometry (Å, °)

*D*—H⋯*A*	*D*—H	H⋯*A*	*D*⋯*A*	*D*—H⋯*A*
C18—H18⋯O2^i^	0.93	2.50	3.395 (3)	163
C44—H44⋯O1^ii^	0.93	2.47	3.369 (3)	161

Symmetry codes: (i) 
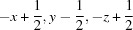
; (ii) 
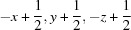
.

**Fig. 1.**
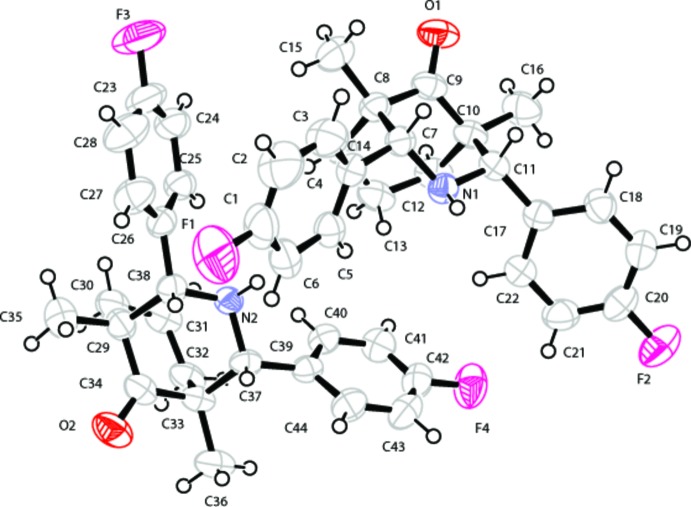
The molecular structure of the title compound, showing displacement ellipsoids at the 50% probability level. H atoms are represented by circles of arbitrary radii.

**Fig. 2.**
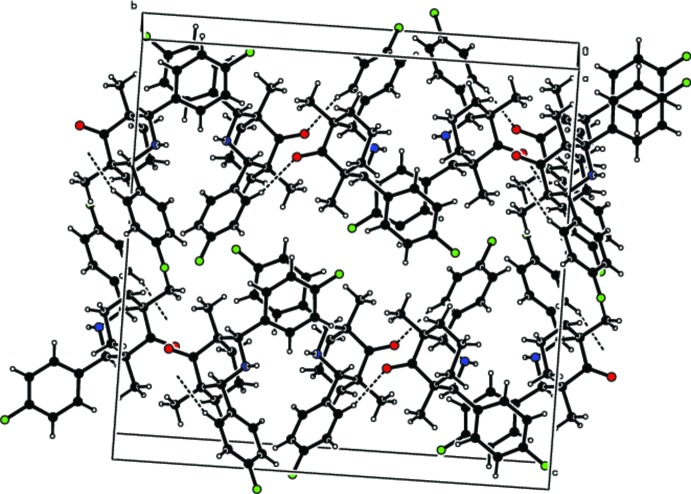
Packing diagram of the title compound.

## supplementary crystallographic information

### 1. Comment

In a continuation of structural studies of 1,5-dimethyl-3-azabicyclo[3.3.1]nonan-9-one derivatives (Venkateswaramoorthi *et al.*, 2013), herewith we present the title compound, (I).

Figure 1 shows the asymmetric unit, which consists of two molecules *A* and *B* of the title compound (I). The bicyclo ring adopts the twin-chair conformation in both molecules, with puckering parameters Q = 0.598 (2) Å, θ = 177.73 (19)° and φ = 37 (4)° for the piperidine ring N1/C7···C11, and Q = 0.548 (2) Å, θ = 11.9 (2)° and φ = 61.6 (11)° for the cyclohexanone ring C8···C14 in molecule *A*. Corresponding parameters for molecule *B* are Q = 0.605 (2) Å, θ = 177.12 (19)° and φ = 326 (4)° for the piperidine N2/C37/C33/C34/C29/C38 and Q = 0.543 (2) Å, θ=11.2 (2)°, and φ = 299.7 (12)° for the cyclohexanone ring C29···C34 (Cremer & Pople, 1975).

The two fluorophenyl rings substituting positions at C7 and C11 in molecule *A* and at C37 and C38 in molecule *B* are planar and are oriented at an angle of 55.27 (8)° and 56.37 (7)° to each other, respectively. In molecule *A*, the piperidine ring makes a dihedral angle of 74.37 (6)° with the fluorophenyl ring C17···C22 and 64.37 (6)° with the fluorophenyl ring C1···C6, whereas in molecule *B*, the corresponding angles are 64.71 (6)° and 74.90 (6)° with the fluorophenyl rings C23···C28 and C39···C44, respectively.

The methyl groups attached to the piperidine ring at C8 and C10 in molecule *A* are in equatorial orientation with torsion angles of -174.75 (16)° (N1—C7—C8—C15) and 176.82 (16)° (N1—C11—C10—C16) respectively. In molecule *B* methyl groups attached to the piperidine ring at C29 and C33 are also in equatorial orientation, with torsion angles of 174.67 (6)° (N2—C38—C29—C35) and -177.52 (16)° (N2—C37—C33—C36).

In the crystal structure of a closely related structure (Venkateswaramoorthi *et al.*, 2012), NH groups are involved in hydrogen bonding, but in the present case, the steric hindrance of fluorophenyl groups avoids the formation of these expected hydrogen bonds. Such a situation was previously reported in the same bicyclic system substituted by difluorophenyl rings (Pham *et al.*, 1998)·In the asymmetric unit of the title compound, atom C18 of molecule *A* acts as a donor for a weak intermolecular interaction. In molecule *B*, C44 acts as a donor for a weak C—H···O interaction *via* H44 with O1 of an adjacent molecule (Table 1, Fig. 2).

### 2. Experimental

Dry ammonium acetate (0.05 mol) was dissolved in ethanol (50 ml) and the solution was mixed with 4-fluoro-benzaldehyde (0.1 mol) and 2,6-dimethyl-cyclohexanone (0.05 mol). The mixture was first refluxed and then allowed to stand at room temperature overnight. Conc. HCl (30 ml) was added and the hydrochloride salt was collected and washed with a mixture of ethanol and ether (1:5 ratio). A suspension of the hydrochloride salt in acetone was treated with strong liquid ammonia solution and the free base was obtained by pouring water. The product was recrystallized from ethanol.

### 3. Refinement

The methyl H atoms were constrained to an ideal geometry (C—H = 0.96 Å) with *U*_iso_(H) = 1.5*U*_eq_(C), but methyl groups were allowed to rotate freely about the C—C bonds. All remaining H atoms bonded to C atoms were placed in geometrically idealized positions (C—H = 0.93–0.98 Å) and constrained to ride on their parent atoms with *U*_iso_(H) = 1.2*U*_eq_(C). N1—H1A and N2—H2A bond lengths were constrained to 0.85 Å using *DFIX*, and bond length C33—C34 was restrained using *DELU* restraint (Sheldrick, 2008).

### Crystal data

**Table:**

C_22_H_23_F_2_NO	*F*(000) = 1504
*M**_r_* = 355.41	*D*_x_ = 1.272 Mg m^−^^3^
Monoclinic, *P*2_1_/*n*	Mo *K*α radiation, λ = 0.71073 Å
Hall symbol: -P 2yn	Cell parameters from 6655 reflections
*a* = 8.8470 (3) Å	θ = 2.5–24.4°
*b* = 20.5656 (8) Å	µ = 0.09 mm^−^^1^
*c* = 20.6403 (9) Å	*T* = 293 K
β = 98.633 (2)°	Block, colourless
*V* = 3712.8 (3) Å^3^	0.35 × 0.35 × 0.30 mm
*Z* = 8	

### Data collection

**Table:**

Bruker Kappa APEXII CCD diffractometer	6363 independent reflections
Radiation source: fine-focus sealed tube	3974 reflections with *I* > 2σ(*I*)
Graphite monochromator	*R*_int_ = 0.038
ω and φ scan	θ_max_ = 24.8°, θ_min_ = 2.2°
Absorption correction: multi-scan (*SADABS*; Bruker, 2004)	*h* = −9→10
*T*_min_ = 0.969, *T*_max_ = 0.973	*k* = −24→23
32409 measured reflections	*l* = −23→24

### Refinement

**Table:**

Refinement on *F*^2^	Primary atom site location: structure-invariant direct methods
Least-squares matrix: full	Secondary atom site location: difference Fourier map
*R*[*F*^2^ > 2σ(*F*^2^)] = 0.043	Hydrogen site location: inferred from neighbouring sites
*wR*(*F*^2^) = 0.125	H-atom parameters constrained
*S* = 1.01	*w* = 1/[σ^2^(*F*_o_^2^) + (0.0508*P*)^2^ + 0.8947*P*] where *P* = (*F*_o_^2^ + 2*F*_c_^2^)/3
6363 reflections	(Δ/σ)_max_ < 0.001
473 parameters	Δρ_max_ = 0.12 e Å^−^^3^
3 restraints	Δρ_min_ = −0.28 e Å^−^^3^
0 constraints	

### Fractional atomic coordinates and isotropic or equivalent isotropic displacement parameters (Å^2^)

**Table:**

	*x*	*y*	*z*	*U*_iso_*/*U*_eq_	
F1	0.3516 (2)	0.48852 (8)	0.45379 (8)	0.1227 (6)	
F2	−0.35332 (15)	0.33318 (8)	−0.02536 (6)	0.0885 (5)	
O1	0.26343 (19)	0.11184 (7)	0.25466 (8)	0.0730 (5)	
N1	0.10400 (16)	0.28936 (7)	0.23517 (7)	0.0444 (4)	
H1A	0.0317	0.3143	0.2435	0.053*	
C1	0.3167 (3)	0.43528 (14)	0.41555 (13)	0.0789 (7)	
C2	0.2802 (4)	0.37929 (15)	0.44484 (12)	0.0948 (9)	
H2	0.2818	0.3775	0.4900	0.114*	
C3	0.2407 (3)	0.32503 (12)	0.40602 (11)	0.0754 (7)	
H3	0.2142	0.2867	0.4255	0.090*	
C4	0.2399 (2)	0.32674 (10)	0.33899 (9)	0.0481 (5)	
C5	0.2782 (2)	0.38521 (10)	0.31226 (10)	0.0519 (5)	
H5	0.2782	0.3878	0.2673	0.062*	
C6	0.3162 (2)	0.43973 (11)	0.35009 (12)	0.0634 (6)	
H6	0.3408	0.4786	0.3311	0.076*	
C7	0.1916 (2)	0.26798 (9)	0.29718 (9)	0.0456 (5)	
H7	0.1232	0.2421	0.3201	0.055*	
C8	0.3259 (2)	0.22271 (10)	0.28393 (10)	0.0515 (5)	
C9	0.2534 (2)	0.16905 (10)	0.23956 (10)	0.0521 (5)	
C10	0.1673 (2)	0.19176 (9)	0.17453 (10)	0.0510 (5)	
C11	0.0377 (2)	0.23670 (9)	0.19285 (9)	0.0457 (5)	
H11	−0.0251	0.2105	0.2182	0.055*	
C12	0.2865 (2)	0.22598 (10)	0.13871 (10)	0.0599 (6)	
H12A	0.3552	0.1932	0.1259	0.072*	
H12B	0.2334	0.2455	0.0989	0.072*	
C13	0.3817 (2)	0.27827 (10)	0.17760 (11)	0.0607 (6)	
H13A	0.4669	0.2896	0.1552	0.073*	
H13B	0.3196	0.3169	0.1794	0.073*	
C14	0.4427 (2)	0.25676 (10)	0.24680 (11)	0.0603 (6)	
H14A	0.4823	0.2946	0.2718	0.072*	
H14B	0.5277	0.2273	0.2451	0.072*	
C15	0.4082 (3)	0.19537 (12)	0.34858 (11)	0.0750 (7)	
H15A	0.4576	0.2301	0.3747	0.113*	
H15B	0.4832	0.1642	0.3398	0.113*	
H15C	0.3353	0.1746	0.3719	0.113*	
C16	0.0966 (3)	0.13486 (10)	0.13357 (11)	0.0728 (7)	
H16A	0.0506	0.1503	0.0913	0.109*	
H16B	0.0200	0.1147	0.1552	0.109*	
H16C	0.1747	0.1037	0.1284	0.109*	
C17	−0.0669 (2)	0.26376 (9)	0.13480 (9)	0.0465 (5)	
C18	−0.2107 (2)	0.23723 (11)	0.11589 (10)	0.0572 (6)	
H18	−0.2424	0.2030	0.1400	0.069*	
C19	−0.3079 (2)	0.26012 (12)	0.06238 (10)	0.0638 (6)	
H19	−0.4038	0.2416	0.0500	0.077*	
C20	−0.2600 (3)	0.31041 (12)	0.02818 (10)	0.0607 (6)	
C21	−0.1216 (3)	0.33934 (11)	0.04500 (11)	0.0658 (6)	
H21	−0.0924	0.3741	0.0209	0.079*	
C22	−0.0253 (2)	0.31588 (10)	0.09870 (10)	0.0579 (6)	
H22	0.0695	0.3354	0.1109	0.070*	
F3	0.8384 (2)	0.25136 (8)	0.46529 (8)	0.1234 (6)	
F4	0.14797 (15)	0.41391 (8)	−0.01577 (6)	0.0864 (4)	
O2	0.77371 (19)	0.62768 (7)	0.26808 (8)	0.0760 (5)	
N2	0.60971 (16)	0.45181 (7)	0.24478 (7)	0.0430 (4)	
H2A	0.5332	0.4288	0.2510	0.052*	
C23	0.8064 (3)	0.30496 (14)	0.42688 (14)	0.0798 (8)	
C24	0.8106 (2)	0.30013 (12)	0.36180 (12)	0.0683 (6)	
H24	0.8360	0.2611	0.3435	0.082*	
C25	0.7762 (2)	0.35463 (10)	0.32321 (10)	0.0533 (5)	
H25	0.7785	0.3519	0.2784	0.064*	
C26	0.7385 (2)	0.41299 (10)	0.34974 (9)	0.0491 (5)	
C27	0.7347 (3)	0.41536 (13)	0.41659 (11)	0.0737 (7)	
H27	0.7079	0.4539	0.4355	0.088*	
C28	0.7702 (3)	0.36108 (16)	0.45542 (12)	0.0928 (9)	
H28	0.7692	0.3630	0.5004	0.111*	
C29	0.8324 (2)	0.51620 (10)	0.29605 (10)	0.0528 (5)	
C30	0.9496 (2)	0.48118 (11)	0.26002 (11)	0.0632 (6)	
H30A	1.0365	0.5098	0.2593	0.076*	
H30B	0.9859	0.4429	0.2851	0.076*	
C31	0.8910 (2)	0.46040 (10)	0.19028 (11)	0.0607 (6)	
H31A	0.9770	0.4483	0.1687	0.073*	
H31B	0.8264	0.4224	0.1911	0.073*	
C32	0.8009 (2)	0.51366 (10)	0.15116 (10)	0.0586 (6)	
H32A	0.7483	0.4949	0.1109	0.070*	
H32B	0.8724	0.5456	0.1392	0.070*	
C33	0.6818 (2)	0.54914 (9)	0.18608 (10)	0.0509 (5)	
C34	0.7642 (2)	0.57082 (10)	0.25176 (10)	0.0526 (5)	
C35	0.9126 (3)	0.54282 (12)	0.36123 (12)	0.0815 (8)	
H35A	0.9606	0.5078	0.3873	0.122*	
H35B	0.8390	0.5636	0.3841	0.122*	
H35C	0.9887	0.5739	0.3533	0.122*	
C36	0.6161 (3)	0.60670 (10)	0.14462 (11)	0.0722 (7)	
H36A	0.5679	0.5914	0.1026	0.108*	
H36B	0.6970	0.6363	0.1388	0.108*	
H36C	0.5421	0.6287	0.1663	0.108*	
C37	0.5485 (2)	0.50556 (9)	0.20268 (9)	0.0462 (5)	
H37	0.4866	0.5323	0.2280	0.055*	
C38	0.6949 (2)	0.47220 (9)	0.30777 (9)	0.0459 (5)	
H38	0.6262	0.4987	0.3300	0.055*	
C39	0.4430 (2)	0.48029 (9)	0.14371 (9)	0.0458 (5)	
C40	0.4816 (2)	0.42821 (10)	0.10662 (10)	0.0538 (5)	
H40	0.5755	0.4078	0.1183	0.065*	
C41	0.3832 (3)	0.40619 (11)	0.05281 (10)	0.0621 (6)	
H41	0.4102	0.3715	0.0280	0.075*	
C42	0.2457 (3)	0.43633 (12)	0.03683 (10)	0.0606 (6)	
C43	0.2012 (3)	0.48678 (12)	0.07203 (11)	0.0672 (6)	
H43	0.1062	0.5063	0.0602	0.081*	
C44	0.3003 (2)	0.50843 (11)	0.12576 (10)	0.0614 (6)	
H44	0.2708	0.5427	0.1505	0.074*	

### Atomic displacement parameters (Å^2^)

**Table:**

	*U*^11^	*U*^22^	*U*^33^	*U*^12^	*U*^13^	*U*^23^
F1	0.1523 (16)	0.1044 (12)	0.1032 (12)	−0.0192 (12)	−0.0071 (11)	−0.0538 (11)
F2	0.0774 (9)	0.1207 (12)	0.0632 (8)	0.0237 (9)	−0.0029 (7)	0.0172 (8)
O1	0.0889 (12)	0.0423 (9)	0.0887 (11)	0.0065 (8)	0.0163 (9)	0.0150 (8)
N1	0.0424 (9)	0.0423 (9)	0.0484 (9)	0.0052 (8)	0.0068 (7)	−0.0027 (8)
C1	0.0792 (18)	0.0744 (18)	0.0776 (18)	−0.0064 (15)	−0.0067 (14)	−0.0271 (16)
C2	0.127 (2)	0.103 (2)	0.0497 (15)	0.001 (2)	−0.0018 (15)	−0.0170 (16)
C3	0.0994 (19)	0.0731 (17)	0.0522 (14)	−0.0020 (15)	0.0067 (13)	−0.0001 (13)
C4	0.0410 (11)	0.0539 (13)	0.0483 (12)	0.0025 (10)	0.0033 (9)	−0.0014 (10)
C5	0.0436 (11)	0.0558 (13)	0.0565 (12)	0.0011 (10)	0.0082 (10)	−0.0069 (11)
C6	0.0534 (13)	0.0582 (14)	0.0774 (16)	−0.0046 (11)	0.0062 (12)	−0.0146 (13)
C7	0.0419 (11)	0.0467 (11)	0.0489 (11)	0.0007 (10)	0.0094 (9)	0.0038 (9)
C8	0.0445 (11)	0.0478 (12)	0.0619 (13)	0.0068 (10)	0.0070 (10)	0.0077 (10)
C9	0.0529 (13)	0.0405 (12)	0.0673 (14)	0.0055 (10)	0.0235 (11)	0.0065 (11)
C10	0.0615 (13)	0.0371 (11)	0.0563 (12)	0.0020 (10)	0.0148 (10)	−0.0004 (10)
C11	0.0481 (11)	0.0420 (11)	0.0487 (11)	−0.0037 (10)	0.0127 (9)	−0.0004 (9)
C12	0.0695 (14)	0.0523 (13)	0.0637 (13)	0.0094 (12)	0.0288 (12)	0.0020 (11)
C13	0.0585 (13)	0.0516 (13)	0.0794 (15)	0.0017 (11)	0.0343 (12)	0.0076 (12)
C14	0.0427 (11)	0.0543 (13)	0.0861 (16)	0.0048 (10)	0.0169 (11)	−0.0005 (12)
C15	0.0703 (15)	0.0717 (16)	0.0786 (16)	0.0154 (14)	−0.0034 (13)	0.0119 (13)
C16	0.0928 (18)	0.0479 (13)	0.0783 (16)	0.0010 (13)	0.0150 (14)	−0.0132 (12)
C17	0.0512 (12)	0.0435 (12)	0.0454 (11)	0.0005 (10)	0.0089 (9)	−0.0050 (9)
C18	0.0554 (13)	0.0618 (14)	0.0550 (13)	−0.0067 (12)	0.0100 (11)	−0.0001 (11)
C19	0.0497 (13)	0.0835 (17)	0.0570 (13)	−0.0032 (13)	0.0044 (11)	−0.0033 (13)
C20	0.0583 (14)	0.0772 (16)	0.0457 (12)	0.0167 (13)	0.0048 (11)	−0.0001 (12)
C21	0.0730 (16)	0.0634 (15)	0.0616 (14)	0.0024 (13)	0.0120 (12)	0.0158 (12)
C22	0.0580 (13)	0.0523 (13)	0.0620 (13)	−0.0052 (11)	0.0040 (11)	0.0042 (11)
F3	0.1348 (14)	0.1159 (13)	0.1122 (13)	0.0213 (11)	−0.0053 (11)	0.0657 (11)
F4	0.0776 (9)	0.1248 (12)	0.0527 (8)	−0.0198 (9)	−0.0036 (7)	0.0028 (8)
O2	0.0903 (12)	0.0420 (9)	0.0966 (12)	−0.0034 (9)	0.0174 (10)	−0.0131 (9)
N2	0.0409 (9)	0.0418 (9)	0.0468 (9)	−0.0027 (8)	0.0080 (7)	0.0058 (8)
C23	0.0733 (17)	0.0836 (19)	0.0781 (18)	0.0118 (15)	−0.0032 (14)	0.0381 (17)
C24	0.0548 (14)	0.0607 (15)	0.0878 (18)	0.0076 (12)	0.0057 (13)	0.0171 (14)
C25	0.0477 (12)	0.0532 (13)	0.0590 (12)	0.0014 (11)	0.0083 (10)	0.0079 (11)
C26	0.0415 (11)	0.0560 (13)	0.0488 (12)	0.0017 (10)	0.0035 (9)	0.0044 (10)
C27	0.0873 (18)	0.0806 (17)	0.0520 (13)	0.0130 (14)	0.0067 (12)	0.0041 (13)
C28	0.110 (2)	0.115 (2)	0.0508 (15)	0.013 (2)	0.0017 (14)	0.0252 (17)
C29	0.0477 (12)	0.0457 (12)	0.0642 (13)	−0.0023 (10)	0.0056 (10)	−0.0047 (11)
C30	0.0423 (12)	0.0534 (13)	0.0961 (18)	−0.0008 (11)	0.0176 (12)	0.0085 (13)
C31	0.0555 (13)	0.0525 (13)	0.0810 (16)	0.0049 (11)	0.0330 (12)	0.0004 (12)
C32	0.0646 (13)	0.0485 (12)	0.0686 (14)	−0.0055 (11)	0.0290 (11)	0.0030 (11)
C33	0.0588 (13)	0.0355 (11)	0.0607 (11)	0.0025 (10)	0.0166 (9)	0.0050 (9)
C34	0.0498 (12)	0.0413 (12)	0.0700 (12)	−0.0024 (10)	0.0204 (9)	−0.0043 (10)
C35	0.0778 (17)	0.0734 (17)	0.0877 (18)	−0.0141 (14)	−0.0058 (14)	−0.0110 (14)
C36	0.0865 (17)	0.0473 (13)	0.0843 (17)	0.0036 (13)	0.0177 (14)	0.0165 (12)
C37	0.0485 (11)	0.0404 (11)	0.0517 (11)	0.0092 (10)	0.0139 (9)	0.0033 (9)
C38	0.0422 (11)	0.0469 (11)	0.0493 (11)	0.0047 (9)	0.0092 (9)	−0.0022 (10)
C39	0.0486 (12)	0.0459 (12)	0.0439 (11)	0.0037 (10)	0.0096 (9)	0.0084 (9)
C40	0.0547 (13)	0.0522 (13)	0.0540 (12)	0.0071 (11)	0.0068 (10)	0.0021 (11)
C41	0.0694 (15)	0.0648 (14)	0.0530 (13)	−0.0005 (13)	0.0124 (11)	−0.0028 (11)
C42	0.0565 (14)	0.0826 (17)	0.0421 (12)	−0.0111 (13)	0.0051 (11)	0.0106 (12)
C43	0.0515 (13)	0.0869 (18)	0.0611 (14)	0.0112 (13)	0.0022 (11)	0.0126 (14)
C44	0.0590 (14)	0.0632 (14)	0.0625 (14)	0.0127 (12)	0.0110 (11)	0.0019 (12)

### Geometric parameters (Å, º)

**Table:**

F1—C1	1.358 (3)	F3—C23	1.363 (3)
F2—C20	1.359 (2)	F4—C42	1.363 (2)
O1—C9	1.217 (2)	O2—C34	1.216 (2)
N1—C11	1.458 (2)	N2—C37	1.459 (2)
N1—C7	1.461 (2)	N2—C38	1.463 (2)
N1—H1A	0.8567	N2—H2A	0.8518
C1—C6	1.354 (3)	C23—C24	1.353 (3)
C1—C2	1.362 (4)	C23—C28	1.356 (4)
C2—C3	1.388 (3)	C24—C25	1.382 (3)
C2—H2	0.9300	C24—H24	0.9300
C3—C4	1.383 (3)	C25—C26	1.381 (3)
C3—H3	0.9300	C25—H25	0.9300
C4—C5	1.386 (3)	C26—C27	1.386 (3)
C4—C7	1.509 (3)	C26—C38	1.510 (3)
C5—C6	1.379 (3)	C27—C28	1.383 (3)
C5—H5	0.9300	C27—H27	0.9300
C6—H6	0.9300	C28—H28	0.9300
C7—C8	1.565 (3)	C29—C34	1.515 (3)
C7—H7	0.9800	C29—C35	1.525 (3)
C8—C9	1.514 (3)	C29—C30	1.542 (3)
C8—C15	1.528 (3)	C29—C38	1.564 (3)
C8—C14	1.544 (3)	C30—C31	1.516 (3)
C9—C10	1.514 (3)	C30—H30A	0.9700
C10—C16	1.522 (3)	C30—H30B	0.9700
C10—C12	1.545 (3)	C31—C32	1.515 (3)
C10—C11	1.563 (3)	C31—H31A	0.9700
C11—C17	1.506 (3)	C31—H31B	0.9700
C11—H11	0.9800	C32—C33	1.546 (3)
C12—C13	1.519 (3)	C32—H32A	0.9700
C12—H12A	0.9700	C32—H32B	0.9700
C12—H12B	0.9700	C33—C34	1.507 (3)
C13—C14	1.515 (3)	C33—C36	1.524 (3)
C13—H13A	0.9700	C33—C37	1.560 (3)
C13—H13B	0.9700	C35—H35A	0.9600
C14—H14A	0.9700	C35—H35B	0.9600
C14—H14B	0.9700	C35—H35C	0.9600
C15—H15A	0.9600	C36—H36A	0.9600
C15—H15B	0.9600	C36—H36B	0.9600
C15—H15C	0.9600	C36—H36C	0.9600
C16—H16A	0.9600	C37—C39	1.510 (3)
C16—H16B	0.9600	C37—H37	0.9800
C16—H16C	0.9600	C38—H38	0.9800
C17—C18	1.385 (3)	C39—C44	1.387 (3)
C17—C22	1.386 (3)	C39—C40	1.389 (3)
C18—C19	1.377 (3)	C40—C41	1.381 (3)
C18—H18	0.9300	C40—H40	0.9300
C19—C20	1.356 (3)	C41—C42	1.360 (3)
C19—H19	0.9300	C41—H41	0.9300
C20—C21	1.359 (3)	C42—C43	1.359 (3)
C21—C22	1.380 (3)	C43—C44	1.380 (3)
C21—H21	0.9300	C43—H43	0.9300
C22—H22	0.9300	C44—H44	0.9300
			
C11—N1—C7	114.47 (14)	C37—N2—C38	114.07 (14)
C11—N1—H1A	108.6	C37—N2—H2A	106.2
C7—N1—H1A	108.6	C38—N2—H2A	109.9
C6—C1—F1	119.5 (3)	C24—C23—C28	122.5 (2)
C6—C1—C2	122.3 (2)	C24—C23—F3	118.9 (3)
F1—C1—C2	118.2 (2)	C28—C23—F3	118.5 (3)
C1—C2—C3	118.7 (2)	C23—C24—C25	118.4 (2)
C1—C2—H2	120.7	C23—C24—H24	120.8
C3—C2—H2	120.7	C25—C24—H24	120.8
C4—C3—C2	121.4 (2)	C26—C25—C24	121.4 (2)
C4—C3—H3	119.3	C26—C25—H25	119.3
C2—C3—H3	119.3	C24—C25—H25	119.3
C3—C4—C5	117.1 (2)	C25—C26—C27	118.0 (2)
C3—C4—C7	120.70 (19)	C25—C26—C38	121.97 (18)
C5—C4—C7	122.15 (17)	C27—C26—C38	120.01 (19)
C6—C5—C4	122.2 (2)	C28—C27—C26	120.7 (2)
C6—C5—H5	118.9	C28—C27—H27	119.6
C4—C5—H5	118.9	C26—C27—H27	119.6
C1—C6—C5	118.3 (2)	C23—C28—C27	118.9 (2)
C1—C6—H6	120.8	C23—C28—H28	120.5
C5—C6—H6	120.8	C27—C28—H28	120.5
N1—C7—C4	109.10 (15)	C34—C29—C35	110.88 (17)
N1—C7—C8	110.05 (15)	C34—C29—C30	106.92 (17)
C4—C7—C8	114.80 (15)	C35—C29—C30	109.61 (18)
N1—C7—H7	107.5	C34—C29—C38	105.89 (15)
C4—C7—H7	107.5	C35—C29—C38	109.93 (17)
C8—C7—H7	107.5	C30—C29—C38	113.53 (16)
C9—C8—C15	111.28 (17)	C31—C30—C29	115.53 (17)
C9—C8—C14	106.53 (17)	C31—C30—H30A	108.4
C15—C8—C14	109.51 (17)	C29—C30—H30A	108.4
C9—C8—C7	105.99 (15)	C31—C30—H30B	108.4
C15—C8—C7	109.93 (17)	C29—C30—H30B	108.4
C14—C8—C7	113.52 (16)	H30A—C30—H30B	107.5
O1—C9—C8	122.8 (2)	C32—C31—C30	112.31 (18)
O1—C9—C10	122.2 (2)	C32—C31—H31A	109.1
C8—C9—C10	114.99 (16)	C30—C31—H31A	109.1
C9—C10—C16	111.42 (17)	C32—C31—H31B	109.1
C9—C10—C12	106.00 (17)	C30—C31—H31B	109.1
C16—C10—C12	109.72 (17)	H31A—C31—H31B	107.9
C9—C10—C11	104.94 (15)	C31—C32—C33	115.72 (16)
C16—C10—C11	109.51 (17)	C31—C32—H32A	108.4
C12—C10—C11	115.13 (16)	C33—C32—H32A	108.4
N1—C11—C17	110.25 (15)	C31—C32—H32B	108.4
N1—C11—C10	109.88 (15)	C33—C32—H32B	108.4
C17—C11—C10	114.30 (15)	H32A—C32—H32B	107.4
N1—C11—H11	107.4	C34—C33—C36	111.57 (17)
C17—C11—H11	107.4	C34—C33—C32	106.72 (17)
C10—C11—H11	107.4	C36—C33—C32	109.52 (17)
C13—C12—C10	115.70 (17)	C34—C33—C37	104.59 (15)
C13—C12—H12A	108.4	C36—C33—C37	109.42 (16)
C10—C12—H12A	108.4	C32—C33—C37	114.94 (15)
C13—C12—H12B	108.4	O2—C34—C33	122.5 (2)
C10—C12—H12B	108.4	O2—C34—C29	122.8 (2)
H12A—C12—H12B	107.4	C33—C34—C29	114.74 (17)
C14—C13—C12	112.48 (17)	C29—C35—H35A	109.5
C14—C13—H13A	109.1	C29—C35—H35B	109.5
C12—C13—H13A	109.1	H35A—C35—H35B	109.5
C14—C13—H13B	109.1	C29—C35—H35C	109.5
C12—C13—H13B	109.1	H35A—C35—H35C	109.5
H13A—C13—H13B	107.8	H35B—C35—H35C	109.5
C13—C14—C8	115.49 (17)	C33—C36—H36A	109.5
C13—C14—H14A	108.4	C33—C36—H36B	109.5
C8—C14—H14A	108.4	H36A—C36—H36B	109.5
C13—C14—H14B	108.4	C33—C36—H36C	109.5
C8—C14—H14B	108.4	H36A—C36—H36C	109.5
H14A—C14—H14B	107.5	H36B—C36—H36C	109.5
C8—C15—H15A	109.5	N2—C37—C39	110.40 (15)
C8—C15—H15B	109.5	N2—C37—C33	109.92 (15)
H15A—C15—H15B	109.5	C39—C37—C33	114.64 (15)
C8—C15—H15C	109.5	N2—C37—H37	107.2
H15A—C15—H15C	109.5	C39—C37—H37	107.2
H15B—C15—H15C	109.5	C33—C37—H37	107.2
C10—C16—H16A	109.5	N2—C38—C26	109.38 (15)
C10—C16—H16B	109.5	N2—C38—C29	109.70 (15)
H16A—C16—H16B	109.5	C26—C38—C29	114.67 (15)
C10—C16—H16C	109.5	N2—C38—H38	107.6
H16A—C16—H16C	109.5	C26—C38—H38	107.6
H16B—C16—H16C	109.5	C29—C38—H38	107.6
C18—C17—C22	117.39 (19)	C44—C39—C40	117.61 (19)
C18—C17—C11	120.46 (18)	C44—C39—C37	119.94 (18)
C22—C17—C11	122.14 (18)	C40—C39—C37	122.44 (18)
C19—C18—C17	121.9 (2)	C41—C40—C39	121.3 (2)
C19—C18—H18	119.1	C41—C40—H40	119.4
C17—C18—H18	119.1	C39—C40—H40	119.4
C20—C19—C18	118.2 (2)	C42—C41—C40	118.6 (2)
C20—C19—H19	120.9	C42—C41—H41	120.7
C18—C19—H19	120.9	C40—C41—H41	120.7
C19—C20—C21	122.8 (2)	C43—C42—C41	122.6 (2)
C19—C20—F2	119.1 (2)	C43—C42—F4	118.9 (2)
C21—C20—F2	118.1 (2)	C41—C42—F4	118.4 (2)
C20—C21—C22	118.5 (2)	C42—C43—C44	118.4 (2)
C20—C21—H21	120.8	C42—C43—H43	120.8
C22—C21—H21	120.8	C44—C43—H43	120.8
C21—C22—C17	121.3 (2)	C43—C44—C39	121.5 (2)
C21—C22—H22	119.3	C43—C44—H44	119.2
C17—C22—H22	119.3	C39—C44—H44	119.2
			
C6—C1—C2—C3	−0.2 (4)	C28—C23—C24—C25	−0.1 (4)
F1—C1—C2—C3	178.4 (2)	F3—C23—C24—C25	179.3 (2)
C1—C2—C3—C4	0.9 (4)	C23—C24—C25—C26	0.1 (3)
C2—C3—C4—C5	−0.9 (3)	C24—C25—C26—C27	−0.6 (3)
C2—C3—C4—C7	−178.0 (2)	C24—C25—C26—C38	−177.63 (17)
C3—C4—C5—C6	0.2 (3)	C25—C26—C27—C28	1.1 (3)
C7—C4—C5—C6	177.27 (17)	C38—C26—C27—C28	178.2 (2)
F1—C1—C6—C5	−179.1 (2)	C24—C23—C28—C27	0.6 (5)
C2—C1—C6—C5	−0.4 (4)	F3—C23—C28—C27	−178.8 (2)
C4—C5—C6—C1	0.4 (3)	C26—C27—C28—C23	−1.1 (4)
C11—N1—C7—C4	−174.22 (14)	C34—C29—C30—C31	−51.5 (2)
C11—N1—C7—C8	58.99 (19)	C35—C29—C30—C31	−171.76 (18)
C3—C4—C7—N1	141.90 (19)	C38—C29—C30—C31	64.9 (2)
C5—C4—C7—N1	−35.1 (2)	C29—C30—C31—C32	45.7 (2)
C3—C4—C7—C8	−94.1 (2)	C30—C31—C32—C33	−45.8 (2)
C5—C4—C7—C8	89.0 (2)	C31—C32—C33—C34	51.7 (2)
N1—C7—C8—C9	−54.38 (19)	C31—C32—C33—C36	172.57 (18)
C4—C7—C8—C9	−177.91 (16)	C31—C32—C33—C37	−63.8 (2)
N1—C7—C8—C15	−174.75 (16)	C36—C33—C34—O2	0.5 (3)
C4—C7—C8—C15	61.7 (2)	C32—C33—C34—O2	120.1 (2)
N1—C7—C8—C14	62.2 (2)	C37—C33—C34—O2	−117.7 (2)
C4—C7—C8—C14	−61.3 (2)	C36—C33—C34—C29	179.78 (17)
C15—C8—C9—O1	−0.9 (3)	C32—C33—C34—C29	−60.6 (2)
C14—C8—C9—O1	118.4 (2)	C37—C33—C34—C29	61.6 (2)
C7—C8—C9—O1	−120.4 (2)	C35—C29—C34—O2	−0.5 (3)
C15—C8—C9—C10	179.21 (17)	C30—C29—C34—O2	−119.9 (2)
C14—C8—C9—C10	−61.5 (2)	C38—C29—C34—O2	118.7 (2)
C7—C8—C9—C10	59.7 (2)	C35—C29—C34—C33	−179.79 (17)
O1—C9—C10—C16	0.9 (3)	C30—C29—C34—C33	60.8 (2)
C8—C9—C10—C16	−179.25 (17)	C38—C29—C34—C33	−60.6 (2)
O1—C9—C10—C12	−118.4 (2)	C38—N2—C37—C39	−171.41 (14)
C8—C9—C10—C12	61.4 (2)	C38—N2—C37—C33	61.16 (19)
O1—C9—C10—C11	119.3 (2)	C34—C33—C37—N2	−57.88 (19)
C8—C9—C10—C11	−60.8 (2)	C36—C33—C37—N2	−177.52 (16)
C7—N1—C11—C17	172.46 (14)	C32—C33—C37—N2	58.8 (2)
C7—N1—C11—C10	−60.68 (19)	C34—C33—C37—C39	177.08 (16)
C9—C10—C11—N1	57.12 (19)	C36—C33—C37—C39	57.4 (2)
C16—C10—C11—N1	176.82 (16)	C32—C33—C37—C39	−66.2 (2)
C12—C10—C11—N1	−59.0 (2)	C37—N2—C38—C26	174.22 (14)
C9—C10—C11—C17	−178.34 (16)	C37—N2—C38—C29	−59.20 (19)
C16—C10—C11—C17	−58.6 (2)	C25—C26—C38—N2	35.1 (2)
C12—C10—C11—C17	65.5 (2)	C27—C26—C38—N2	−141.86 (19)
C9—C10—C12—C13	−51.8 (2)	C25—C26—C38—C29	−88.6 (2)
C16—C10—C12—C13	−172.27 (18)	C27—C26—C38—C29	94.4 (2)
C11—C10—C12—C13	63.7 (2)	C34—C29—C38—N2	54.84 (19)
C10—C12—C13—C14	46.1 (2)	C35—C29—C38—N2	174.67 (16)
C12—C13—C14—C8	−45.7 (2)	C30—C29—C38—N2	−62.1 (2)
C9—C8—C14—C13	51.5 (2)	C34—C29—C38—C26	178.38 (16)
C15—C8—C14—C13	171.97 (18)	C35—C29—C38—C26	−61.8 (2)
C7—C8—C14—C13	−64.8 (2)	C30—C29—C38—C26	61.4 (2)
N1—C11—C17—C18	−133.43 (18)	N2—C37—C39—C44	131.91 (18)
C10—C11—C17—C18	102.2 (2)	C33—C37—C39—C44	−103.3 (2)
N1—C11—C17—C22	46.0 (2)	N2—C37—C39—C40	−46.7 (2)
C10—C11—C17—C22	−78.4 (2)	C33—C37—C39—C40	78.1 (2)
C22—C17—C18—C19	1.7 (3)	C44—C39—C40—C41	1.6 (3)
C11—C17—C18—C19	−178.85 (18)	C37—C39—C40—C41	−179.81 (18)
C17—C18—C19—C20	−0.5 (3)	C39—C40—C41—C42	−0.5 (3)
C18—C19—C20—C21	−0.8 (3)	C40—C41—C42—C43	−0.6 (3)
C18—C19—C20—F2	179.09 (18)	C40—C41—C42—F4	−179.10 (18)
C19—C20—C21—C22	0.8 (3)	C41—C42—C43—C44	0.5 (3)
F2—C20—C21—C22	−179.04 (19)	F4—C42—C43—C44	179.06 (18)
C20—C21—C22—C17	0.4 (3)	C42—C43—C44—C39	0.6 (3)
C18—C17—C22—C21	−1.6 (3)	C40—C39—C44—C43	−1.6 (3)
C11—C17—C22—C21	178.92 (18)	C37—C39—C44—C43	179.74 (19)

### Hydrogen-bond geometry (Å, º)

**Table:**

*D*—H···*A*	*D*—H	H···*A*	*D*···*A*	*D*—H···*A*
C18—H18···O2^i^	0.93	2.50	3.395 (3)	163
C44—H44···O1^ii^	0.93	2.47	3.369 (3)	161

Symmetry codes: (i) −*x*+1/2, *y*−1/2, −*z*+1/2; (ii) −*x*+1/2, *y*+1/2, −*z*+1/2.
